# Serum phosphate and phosphate-regulatory hormones in COPD patients

**DOI:** 10.1186/s12931-018-0889-6

**Published:** 2018-09-20

**Authors:** Alexandra Stroda, Vincent Brandenburg, Ayham Daher, Christian Cornelissen, Claudia Goettsch, András Keszei, Michael Dreher

**Affiliations:** 1 Department of Pneumology and Intensive Care Medicine, University Hospital Aachen, Aachen, Germany; 2 Department of Cardiology, Angiology and Intensive Care Medicine, University Hospital Aachen, Würselen, Germany; 3 Department of Cardiology, Angiology and Intensive Care Medicine, University Hospital Aachen, Aachen, Germany; 4 Department of Cardiology and Nephrology, Rhein-Maas Klinikum Würselen, Aachen, Germany

**Keywords:** Chronic obstructive pulmonary disease, Fibroblast growth factor 23, Phosphate, Parathyroid hormone

## Abstract

**Background:**

Fibroblast growth factor 23 (FGF23) regulates phosphate metabolism by increasing renal phosphate excretion and decreasing 1.25-dihydroxyvitamin D synthesis. Reports about hypophosphatemia in patients with chronic obstructive pulmonary disease (COPD) suggest altered phosphate metabolism. Therefore, we hypothesized that disturbances in phosphate-regulatory hormones such as FGF23 and parathyroid hormone (PTH) are present in COPD patients.

**Methods:**

We investigated 40 COPD patients (63.5 ± 9.9 years, 27 male), each matched with two age- and sex-matched controls without any primary lung disease. COPD patients underwent lung function testing in advance. All patients had a glomerular filtration rate (GFR) > 60 mL/min/1.73m^2^. We measured concentrations of intact FGF23 (iFGF23) and c-terminal FGF23 (c-term FGF23), phosphate, parathyroid hormone (PTH) and C-reactive protein (CRP) levels in COPD patients and controls.

**Results:**

Phosphate (1.0 ± 02 vs. 1.1 ± 0.2 mmol/L; *p* = 0.027), PTH (54.2 ± 29.4 vs. 68.7 ± 31.8 pg/mL; *p* = 0.002) and iFGF23 (46.3 ± 29.0 vs. 57.5 ± 33.5 pg/mL; *p* = 0.026 ) levels were significantly lower in COPD patients compared with controls. There was a significant negative correlation between c-term FGF23 and total lung capacity (*r* = − 0.4; *p* = 0.01), and between c-term FGF23 and CRP in COPD patients (*r* = 0.48; *p* = 0.002). iFGF23 and c-term FGF23 were positively correlated with phosphate and PTH in the control group.

**Conclusion:**

We confirmed lower average serum phosphate levels in COPD patients compared with controls. However, our data do not suggest a causative role for FGF23 or PTH in COPD because levels of both phosphate-lowering hormones appear to be adaptively decreased as well. Therefore, further investigations are needed to identify the pathogenesis of low phosphate levels in patients with COPD and the relationship between phosphate-regulatory hormones and disease progression.

## Background

Chronic obstructive pulmonary disease (COPD) is a serious condition that is increasing in incidence and is and is one of the leading causes of death worldwide [1]. COPD affects 5–15% of adults in Western countries [2] and it is predicted to cause 4.1% of all deaths in these countries by 2030 [1].

Several lines of evidence indicate that phosphate-fibroblast growth factor-23 (FGF23) signaling pathways deserve attention in patients with COPD, in whom a tendency for hypophosphatemia has been described [3, 4]. FGF23 is a major regulator of phosphate metabolism leading to increased renal phosphate excretion and diminished synthesis of 1,25-dihydroxyvitamin D [5–7]. Genetic deficiency of FGF23 results in a lethal phenotype in mice, including premature aging and severe pulmonary emphysema [8]. It equals the lethal phenotype of the klotho-knockout mouse, which can be ameliorated by phosphate restriction, showing that phosphate drives a large extent the phenotypic changes characterizing the aging process [9]. Genetic ablation of FGF23 associates in vivo with a phenotype of premature aging but accumulating data points to circulating levels of FGF23 in serum being a potentially independent risk factor for increased cardiovascular risk and is associated with elevated cardiovascular and all-cause mortality in various patient cohorts, especially those with chronic kidney and cardiac diseases [10–12].

Preliminary data from earlier studies indicate that COPD is linked with low phosphate levels in both muscles and the circulation [3, 4]. In addition, hypophosphatemia predicted poor outcome in hospitalized COPD patients [13]. Only one study so far, conducted in Saudi Arabia, has addressed a potentially causative relation between FGF23 and COPD. However, this study has several limitations, including the exclusion and inclusion criteria, use of concomitant medications, and glomerular filtration rate (GFR) as confounding factor [14]. Thus, existing data are limited and cannot establish a clear cause-and-effect relationship.

We hypothesize that phosphate metabolism and its major regulator FGF23 play a role in COPD. Here, we investigated a well-characterized cohort of COPD patients with respect to phosphate metabolism status, including FGF23 and parathyroid hormone (PTH), and compared these results with age- and sex-matched controls without COPD. The aim was to determine whether COPD patients have relevant changes in serum phosphate levels and phosphate-regulatory hormones or factors.

## Methods

This cross-sectional case-control study was conducted at the University Hospital RWTH Aachen, Aachen, Germany. The study protocol was approved by the local ethics committee (190/50,07-22-2015). Blood samples were taken from November 2015 until June 2017. All patients signed informed consent prior to the study. The implemented methods are in accordance with the latest revision of the Declaration of Helsinki.

### Participants

COPD patients underwent a lung function test, and were then assigned two sex- and aged- matched controls who did not have COPD or any other primary lung disease. Exclusion criteria for COPD patients were a GFR < 60 mL/min/1.73 m^2^ and any other primary pulmonary diseases except for COPD. Exclusion criteria for control patients were a GFR < 60 mL/min/1.73 m^2^ or any primary pulmonary disease. Gender, age, height, weight and body mass index (BMI) were recorded for all participants.

### Lung function testing

All but one of the COPD patients had previously undergone whole-body plethysmography (the remaining patient had spirometry). All COPD patients were classified according to the Global Initiative for Chronic Obstructive Lung Disease (GOLD) guidelines [15].

### Measurements and laboratory values

iFGF23 and c-term FGF23 concentrations in plasma were measured using a two-site enzyme-linked immunosorbent assay (ELISA) kit (Immutopics, Inc., 96 Test Kit, Cat. # 60–6600 for iFGF23 and Immutopics, Inc., 96 Test Kit, Cat. # 60–6100 for c-term FGF23) according to the manufacture protocol [16]. To detect the amino-terminal and carboxyl-terminal portions of iFGF23, two antibodies were used: a murine monoclonal antibody, which was biotinylated for capture and an affinity purified goat polyclonal antibody conjugated with the enzyme horseradish peroxidase (HRP) for detection. As instructed, 50 μL of standard, control and sample were pipetted into the streptavidin-coated microtiter well followed by 50 μL of biotinylated antibody. After incubation and washing five times, 100 μL of HRP-conjugated human FGF23 antibody was pipetted into the wells. The well was then incubated and washed another five times. 100 μL of ELISA HRP substrate was added followed by incubation. Absorbance was read before (OD_595nm_) and after (OD_450nm_) adding 50 μL of Stop Solution. The detection is limit 1.5 pg/mL. The intra- and interassay coefficients of variation were 2.0–4.1% and 3.5–9.1%, respectively.

C-term FGF23 was assayed in a similar way. However, in this assay the biotinylated capture antibody was also an affinity-purified goat antibody. The used antibodies only detected epitopes within the c-term portion of FGF23 and bound to the intact molecule and the large carboxyl terminal fragments of human FGF23. As per the instructions, 100 μL of standard, control and sample were pipetted into streptavidin-coated wells. Afterwards, 50 μL of Working Antibody Solution, containing both antibodies, was added. After incubation, the wells were washed five times and 150 μL of ELISA HRP Substrate was added. The remaining steps were as described above. The detection is limit 1.5 RU/mL. The intra- and interassay coefficients of variation were 1.4–2.4% and 2.4–4.7%, respectively.

All samples were assayed in duplicate. Serum concentrations of C-reactive protein (CRP), phosphate, plasma PTH, GFR, creatinine, urea and hemoglobin (Hb) were quantified using standardized laboratory procedures implemented by the laboratory diagnostic center (LDZ) of the university hospital Aachen.

### Statistical analysis

Statistical analysis was performed using the SPSS 25.0 statistical package (IBM Corp. Released 2017. IBM SPSS Statistics for Windows, Version 25.0. Armonk, NY: IBM Corp.) and R (R Core Team (2016). R: A language and environment for statistical computing. R Foundation for Statistical Computing, Vienna, Austria.).

Baseline characteristics, laboratory values and lung function parameters are presented as mean ± standard deviation (SD) with *p*-values calculated using a Student’s t-test for normally distributed data. All skewed continuous parameters are also presented as median (quartile [Q] 1 – Q3) with p-values determined using a Mann-Whitney U-test for non-normally distributed data. *p*-values of < 0.05 were considered statistically significant.

IFGF23 and c-term FGF23 level data were statistically analyzed using a logarithmic transformation of iFGF23 and c-term FGF23 concentrations. We designed a linear mixed model with fixed effects for case-control status and random effects of matched groups and subjects. The logarithmic transformations of iFGF23 and c-term FGF23 were maintained during correlation analysis using Pearson’s correlation coefficient. We investigated correlations between FGF23 and lung function parameters in the COPD group by determining Pearson’s correlation coefficient due to approximate linear regression seen in the scatterplots. Correlations between FGF23 and phosphate, PTH and CRP were determined using Spearman’s correlation coefficient, because we could not determine linear correlation between these parameters using the scatterplot.

## Results

### Baseline characteristics, laboratory values, lung function

A total of 119 participants were enrolled in the study (40 patients with COPD and 79 controls) (Table 1). Fifty-one of the control subjects had no COPD based on the GOLD classification or whole-body plethysmography; COPD was not mentioned in medical records for the 28 control group subjects who did not have a lung function test during hospitalization. One control subject was excluded from the analysis because of a previous blood transfusion. Patients with COPD were in GOLD stage I (*n* = 3, 7.5%), II (*n* = 13, 32.5%), III (*n* = 10, 25%) or IV (*n* = 14, 35%).

**Table 1:** Baseline characteristics, laboratory values and lung function parameters for patients and controls

	COPD patients (*n* = 40)	Controls (*n* = 79)	*p*-value
Male sex, n (%)	27 (67.5)	54 (68.4)	
Age, years	63.5 ± 9.9	63.7 ± 9.8	0.908
Height, m	1.7 ± 0.1	1.7 ± 0.1	0.598
Weight, kg	83.2 ± 19.2	84.9 ± 16.5	0.624
Body mass index, kg/m^2^	27.3 ± 5.9	28.3 ± 5.1	0.387
GFR, mL/min/1.73m^2^	88.9 ± 11.3	82.7 ± 12.9	0.026
Creatinine, mg/dL	0.8 ± 0.2	0.9 ± 0.2	0.022
Urea, mg/dL^a^	30.1 ± 8.8	35.5 ± 14.2	0.013
Hemoglobin, g/dL	13.6 ± 1.5	13.0 ± 1.8	0.058
Lung function^b^			
FEV_1_, L	1.4 ± 0.8	2.6 ± 0,8	< 0.0001
FEV_1_, % predicted	47.5 ± 22.5	87.3 ± 17.6	< 0.0001
FEV_1_/FVC ratio, %	49.8 ± 14.2	80.6 ± 6.6	< 0.0001
TLC, L	7.7 ± 1.7	6.1 ± 1.5	< 0.0001
TLC, % predicted	118.7 ± 17.5	95.4 ± 18.1	< 0.0001
RV, L	4.9 ± 1.7	2.8 ± 0.9	< 0.0001
RV, % predicted	208.1 ± 62.5	123.0 ± 39.5	< 0.0001
VC, L	2.8 ± 1.0	3.3 ± 0.9	0.021
VC, % predicted	71.6 ± 21.4	84.3 ± 16.4	0.003
R total	0.76 ± 0.46 (0.65; 0.46–0.91)	0.31 ± 0.13 (0.3; 0.25–0.34)	< 0.0001
R total, % predicted	252.9 ± 155.2 (216.6; 153.2–302.9)	104.1 ± 43.1 (98.2; 84.4–114.9)	< 0.0001

^a^Urea data available for 39 COPD patients; ^b^Overall, lung function was assessed in 51 patients from the control group; data for TLC, RV, VC and R were available for 39 patients with COPD

Values are number of patients (%) or mean ± SD, with median; Q1-Q3 values shown for skewed continuous parameters

FEV_1_, forced expiratory volume in 1 s; *Q* quartile; *R* resistance; *RV* residual volume; *VC* vital capacity.

There were no significant differences in sex, height, weight and BMI between COPD patients and controls (Table 1). Although GFR differed significantly between the two groups (Table 1), all patients could be clinically classified as chronic kidney disease stage I-II (CKD I-II) [17]. Lung function was significantly impaired in COPD patients versus controls (Table 1). Coronary heart disease (CHD) was present in 17 patients with COPD and 57 controls.

### iFGF23 and c-term FGF23 concentrations

Table 2 shows concentrations of iFGF23, c-term FGF23, CRP, PTH and phosphate in patients with COPD and the control group. Application of the model suggested that iFGF23 concentration was 27% higher in controls than in COPD patients. The effect estimate on transformed scale for case-control status was 0.24 with a 95% confidence interval (CI) of 0.03–0.45; *p* = 0.026. When the model was adjusted for GFR, the between-group difference remained significant (estimate 0.23; 95% CI 0.01–0.45; *p* = 0.04).

**Table 2:** Laboratory findings and concentrations of serum phosphate and phosphate-regulatory hormones

	COPD patients(*n* = 40)	Controls(*n* = 79*)*	*p*-value
CRP, mg/L	14.3 ± 22.8 (5.9; 1.3–10.6)	14.4 ± 17.4 (7.5; 2.4–20.8)	0.226^a^
Phosphate, mmol/L	1.0 ± 0.2	1.1 ± 0.2	0.027^b^
PTH, pg/mL	54.2 ± 29.4 (44; 36.4–62.1)	68.7 ± 31.8 (61.1; 46.9–83.3)	0.002^a^
iFGF23, pg/mL	46.3 ± 29.0 (38.9; 29.0–55.9)	57.5 ± 33.5 (51.9; 34.3–71.7)	0.026^c^
c-term FGF23, RU/mL	121.6 ± 191.6 (65.2; 54.2–88.2)	120.6 ± 98.8 (93.2; 55.8–143.8)	0.5^c^

^a^ Mann-Whitney-U. ^b^T-test. ^c^Difference calculated using the model described in the methods section

Values are number of patients (%) or mean ± SD, with median; Q1–Q3 values shown for skewed continuous parameters.

*CRP* C-reactive protein; *c-term FGF23* c-terminal fibroblast growth factor-23; *GFR* glomerular filtration rate; *iFGF23* intact fibroblast growth factor-23; *PTH* parathyroid hormone.

The model representing the results for c-term FGF23 was developed in a similar manner to the one for iFGF23. One specimen showed values > 1478.5 RU/mL and the ELISA kit could not give more exact data. Working with estimators, we could retain a result representing all measured concentrations. The case-control comparison did not show a significant difference of c-term FGF23 in controls compared with COPD patients. The effect estimate (on transformed scale) for case-control status was 0.09 with a 95% CI of − 0.17, 0.36; *p* = 0.494.

### Correlation between iFGF23 and c-term FGF23 and lung function parameters in COPD

**Fig. 1:**
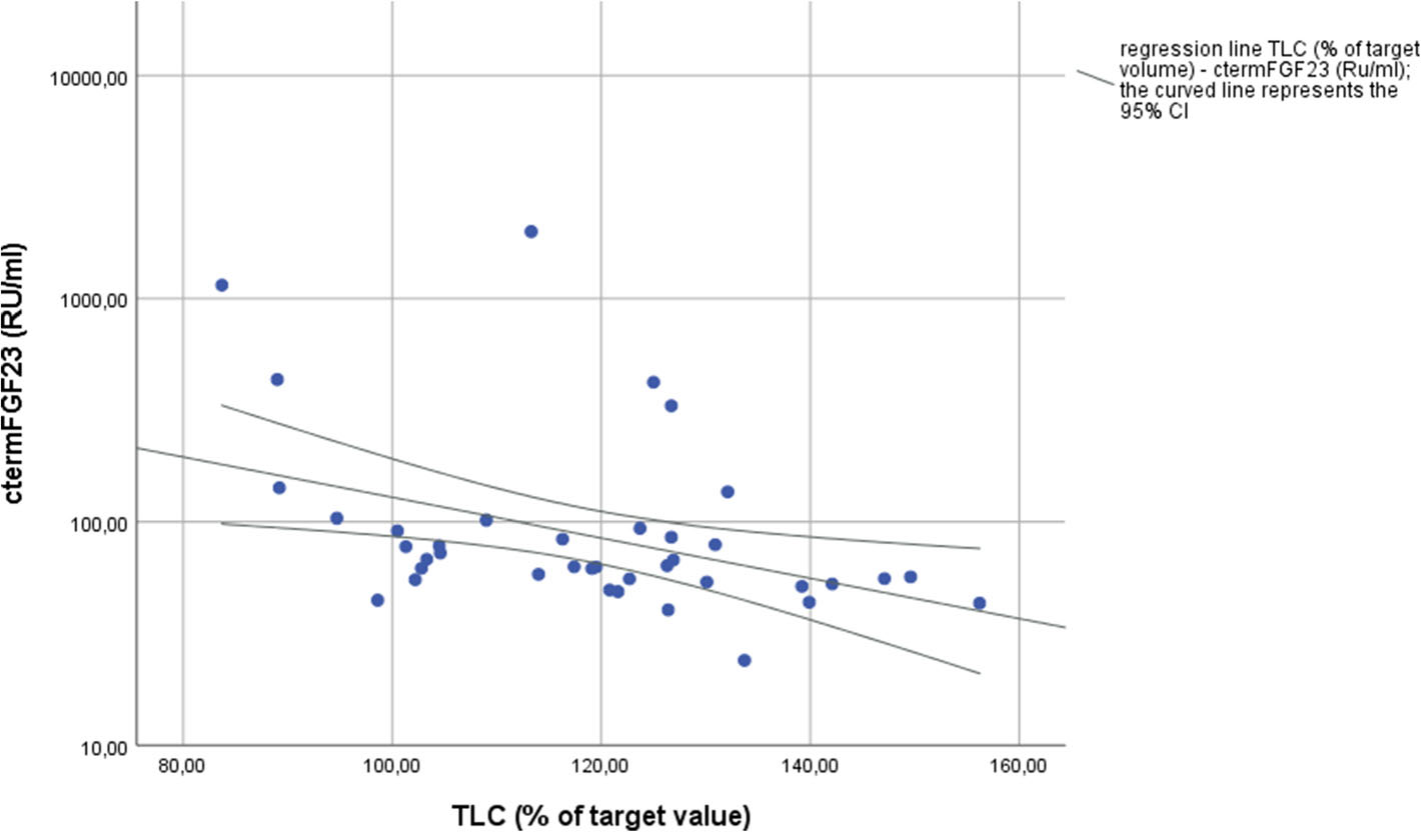
Relationship between total lung capacity (TLC) and c-term FGF23 in chronic obstructive pulmonary disease patients

Scatterplots suggest approximately linear correlations between iFGF23 and FEV_1_/FVC ratio, FEV_1_ and TLC. There was no significant correlation between logarithmic transformed iFGF23 and FEV_1_, FEV_1_/FVC ratio and TLC. Pearson’s correlation coefficient with logarithmic transformed iFGF23 in patients with COPD were as follows: FEV_1_, *r* = − 0.24, *p* = 0.13; FEV_1_/FVC ratio, *r* = − 0.09, *p* = 0.59; TLC, *r* = − 0.22, *p* = 0.18. There was an approximately linear correlation between logarithmic transformed c-term FGF23 and TLC (r = − 0.4, *p* = 0.01) (Fig. 1). The other correlations with logarithmic transformed c-term FGF-23 in cases were: FEV_1_, *r* = − 0.25, *p* = 0.13; FEV_1_/FVC ratio, *r* = − 0.10, *p* = 0.58.

### CRP, phosphate and PTH levels in COPD patients and controls

Hypophosphatemia (phosphate < 0.8 mmol/L) was detected in 12.5% of COPD patients and 5% of controls (*p* = 0.25). There was no significant difference in CRP levels between COPD patients and controls, but levels of PTH and phosphate were significantly lower in COPD patients versus controls (Tab. 2).

### Correlation of CRP, phosphate and PTH with FGF23 in COPD patients and controls

Because there was no distinct linear relationship for PTH and phosphate with iFGF23 and c-term FGF23, Spearman’s correlation coefficients were calculated (Table 3). This was also the case for the correlation between CRP and either iFGF23 or c-term FGF23. In the control group, there was a significant positive correlation for phosphate and PTH with iFGF23 and c-term FGF23. In patients with COPD, there was a significant positive correlation between c-term FGF23 and CRP.

**Table 3:** Correlation coefficients for intact and c-terminal fibroblast growth factor-23 (FGF23) with phosphate and parathyroid hormone (PTH) levels

FGF23	Variable	Group	Correlation coefficient	*p*-value
*intact*	Phosphate	Case	0.3	0.057
*intact*	Phosphate	Control	0.31	0.006
*intact*	PTH	Case	0.29	0.067
*intact*	PTH	Control	0.25	0.02
*c-term*	Phosphate	Case	0.23	0.15
*c-term*	Phosphate	Control	0.29	0.01
*c-term*	PTH	Case	0.3	0.064
*c-term*	PTH	Control	0.27	0.016
*intact*	CRP	Case	0.16	0.31
*intact*	CRP	Control	−0.16	0.157
*c-term*	CRP	Case	0.48	0.002
*c-term*	CRP	Control	0.05	0.683

## Discussion

To the best of our knowledge, this study is the most detailed investigation of disturbed phosphate metabolism in COPD patients to date. We have confirmed that serum phosphate levels are lower in COPD patients, which is consistent with previous studies showing that COPD patients have low phosphate levels in both muscles and the circulation [3, 4]. Our data expand previous findings by providing a sophisticated analysis of important phosphate-regulatory hormones in COPD patients [18], showing that the level of iFGF23 and PTH are reduced in COPD patients compared with controls. This suggests the possibility that the alteration in serum phosphate is the primary modification, which is followed by reductions in phosphate-lowering hormones. What is causing low phosphate levels and the tendency to develop hypophosphatemia in COPD patients cannot be determined from our data. Changes in the two major phosphate regulatory hormones PTH and iFGF23 appear to be adaptive to primary phosphate alterations assuming a physiological feedback loop between phosphate and its regulators.

In contrast to our findings, a previous cross-sectional study conducted in Saudi Arabia partly reported higher FGF23 and PTH levels in COPD patients versus controls [14]. Several aspects differ between the two studies and potentially explain the different results, including the distribution of GOLD stages within the COPD group, ethnicity, and different exclusion and inclusion criteria. The Saudi Arabian study excluded patients taking medications that could affect bone metabolism, but did not focus explicitly on GFR as confounding factor. Moreover, they did not distinguish between c-term FGF23 and iFGF23, and it is not clear which of these has been measured [14]. The disparate findings of these two studies investigating the role of FGF23 in COPD patients highlights the need for further investigations in this field to clearly elucidate the role of FGF23 in COPD patients.

Regarding correlation of both iFGF23 and c-term FGF23 with lung function parameters in humans, we showed a significant moderate negative correlation between the metabolic product c-term FGF23 and TLC. We are the first to show that patients with an abnormal distension of the lung have lower levels of the degradation product c-term FGF23. In our opinion, further investigations are needed to determine whether this is a cause-effect relationship and to identify whether there is a direct effect of FGF23 in the lung tissue.

Earlier studies showed a significant association between inflammation, including CRP, and FGF23 levels [19, 20]. In our study there was no significantly difference in CRP levels between COPD patients and controls, suggesting a similar level of systemic inflammation in the two groups. Therefore, inflammation is unlikely to be a confounder of FGF23 levels in our study.

FGF23 is known to be higher in patients with CKD, especially in severe stages [21]. Therefore, we excluded patients with a GFR < 60 mL/min/1.73 m^2^. This means that our results were not influenced by a low GFR. The only exclusion criteria for the control group was a lack of primary lung diseases, meaning that we included a diverse and representative sample of hospitalized individuals.

The study has several limitations that should be highlighted. Firstly, not all control patients underwent lung function assessment to exclude COPD. Therefore, some control patients may have had undetected COPD. However, lung function testing was performed in the majority of control patients and none had COPD mentioned in previous medical reports. Another limitation was the small sample size in our study. Studies with a larger sample size may be able to detect a statistically significant relationship between iFGF23 and the severity of COPD based on FEV_1_ and TLC. Furthermore, many patients in the control group had CHD, which could have influenced our results because FGF23 is a known risk factor for cardiovascular disease [11]. A confounding factor regarding phosphate levels might be varying medication among the patients potentially contributing to hypophosphatemia. Especially ß2-agonists, corticosteroids, xanthine derivates and hydrochlorothiazide (HCT) are known to influence phosphate homeostasis [22]. Among the controls three persons were medicated with ß2-agonists, three with corticosteroids, none with xanthine derivates and 15 with HCT. Regarding the COPD-patients 29 were medicated with ß2-agonists, 12 with corticosteroids, one with xanthine derivates and eight with HCT. Finally, a potential limitation of our trial is, that we cannot comment on tissue levels of FGF23 and its receptors. Moreover, we have not assessed circulating klotho levels in our studies. The density and distribution of klotho influences FGF23 metabolism and also the FGF23 signalling cascade. However, currently available klotho assays lack specifity and have only limited diagnostic power regarding biological activity of the klotho-FGF23-system [23].

In conclusion, iFGF23 level and concentrations of PTH and phosphate were lower in our cohort of COPD patients compared with non-COPD controls. These data suggest that PTH and FGF23 react to, rather than induce, changes in phosphate homeostasis, which we therefore consider to be the primary defect. Causes of this low serum phosphate remain unknown and we acknowledge that we cannot provide data about phosphate distribution within deeper compartments (e.g. muscles) or intracellularly. Therefore, further investigations are needed including investigations about renal phosphate handling and the role of klotho in this setting.

## Abbreviations

BMI: Body mass index
CHD: Coronary heart disease
CI: Confidence interval
CKD: Chronic kidney disease
COPD: Chronic obstructive pulmonary disease
CRP: C-reactive protein
c-term FGF23: C-terminal fibroblast growth factor 23
ELISA: Enzyme-linked immunosorbent assay
FEV_1_: Forced expiratory volume in 1 s
FGF23: Fibroblast growth factor 23
GFR: Glomerular filtration rate
GOLD: Global Initiative for Chronic Obstructive Lung Disease
Hb: Hemoglobin
HCT: Hydrochlorthiazide
HRP: Horseradish peroxidase
iFGF23: Intact fibroblast growth factor 23
PTH: Parathyroid hormone
Q: Quartile
RV: Residual volume
SD: Standard deviation
TLC: Total lung capacity
VC: Vital capacity
